# Factors Associated With Workplace Bullying and the Mental Health of Construction Industry Apprentices: A Mixed Methods Study

**DOI:** 10.3389/fpsyt.2021.629262

**Published:** 2021-05-14

**Authors:** Victoria Ross, Sharna L. Mathieu, Rachmania Wardhani, Jorgen Gullestrup, Kairi Kõlves

**Affiliations:** ^1^Australian Institute for Suicide Research and Prevention, School of Applied Psychology, Griffith University, Mount Gravatt, QLD, Australia; ^2^MATES in Construction, Spring Hill, QLD, Australia

**Keywords:** workplace bullying, construction industry, apprentices, mental health, well-being, suicidal behaviors

## Abstract

Young Australian males working in the construction industry are twice as likely to take their own lives than other young Australian males. This group is also at high risk for poor mental health and alcohol and other drug related harm. Previous research has indicated a bullying culture within this industry, directed particularly toward apprentices and those new to the industry. This Australian study applied an exploratory sequential mixed methods design to explore issues faced by apprentices, estimate the prevalence of bullying and explore the factors associated with bullying and the mental health of apprentices. The results revealed that a substantial proportion of construction industry apprentices experience workplace bullying, are exposed to suicidal behaviors, and personally experience suicidal ideation. Multivariate analyses showed that bullying in apprentices was significantly associated with greater psychological distress, as well as being a 3rd year apprentice or not currently in an active apprenticeship. Results also indicated that bullying may be associated with substance use, lower levels of well-being, working nights away from home, the plumbing trades, and working for larger organizations. The outcomes from this study have important implications for the construction industry and will be vital for informing policies and evidence-based interventions to address bullying and mental health in this sector.

## Introduction

It is well-established that construction workers represent one of the highest occupational risk groups for suicide internationally (1–6). Elevated suicide rates in this male-dominated industry are understood to be associated with a range of issues such as excessive alcohol consumption, relationship problems, lack of job security and adverse psychosocial working conditions (2, 7). In addition to the stress and physical demands of construction work, the industry culture is dominated by traditional masculine beliefs such as self-reliance and stoicism, which serve as a barrier to help-seeking (8–10). Self-reliance, in particular, has also been positively linked to increased risk of suicidal thinking (11).

In Australia, there is alarming evidence that young males working in the construction industry are twice as likely to take their own lives than are other young Australian males (2). This group is also at high risk for poor mental health and alcohol and other drug related harm (12). The higher suicide rates in younger construction workers are thought to be related to the pressures associated with working within a “masculine” industry which has a bullying culture, particularly directed toward apprentices and those new to the industry (12).

In a qualitative study of Australian construction industry apprentices, participants described a range of workplace bullying experiences, but despite this often-obvious poor treatment, apprentices did not feel able to communicate their distress and commonly described a fear of further adverse outcomes (12). Additionally, the authoritarian organizational culture and paternalistic hierarchies often seen in the construction industry meant that bullying was frequently disguised as “humor” or was systemic to the worksites (12).

For construction apprentices, worksite bullying has been linked to increased substance use (13), poorer mental health/suicidality, and avoidant coping strategies (14), as well as job dissatisfaction and apprenticeship non-completion (15). Workplace bullying may also be associated with toxic ideals of Western masculinity in the traditionally male-dominated industry, with weaker/smaller/younger workers frequently becoming targets (14). Thus, there are a number of factors that may impact the mental health and well-being of construction apprentices; however, research in this area has so far relied upon small qualitative studies and are limited in their generalizability. The current study aimed first, to qualitatively explore the experiences of apprentices and their mental health and well-being. Workplace bullying emerged as a key theme that was associated with a host of negative outcomes (e.g., anxiety of changing worksites, fear of seeking help, stress, etc.); therefore, the second aim was to quantitatively examine the prevalence of workplace bullying and its' associations with key psychosocial variables in this vulnerable group (e.g., substance use, well-being).

## Materials and Methods

**Figure 1:**
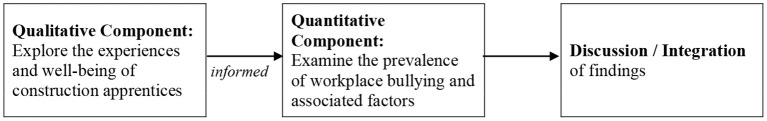
Methodological overview of the exploratory sequential mixed methods design.

The study, set in Queensland, Australia, applied an exploratory sequential mixed methods design (16, 17). An initial qualitative study used a series of focus groups to explore key issues experienced by construction industry apprentices (see Figure 1). The findings from these exploratory results, including the emergence of workplace bullying as a key (yet unprompted) theme, were then used to inform the development of a large-scale quantitative survey to identify the prevalence of bullying, the demographic characteristics of apprentices being bullied, and to understand the impact of bullying and other factors on apprentices' mental health, well-being, and suicidality. The research was conducted in collaboration with Mates in Construction (MATES), which incorporates the knowledge/experiences from lived experience of suicidal behaviors in their early intervention and prevention program designed to reduce suicide in the construction industry.

### Qualitative Study: Focus Groups

The aim of the qualitative study was to explore problems experienced at work and outside of work by construction industry apprentices from their own perspectives, and the types of information and supports that would be helpful in increasing well-being and mental health.

#### Participants and Procedure

Six focus groups were conducted in Queensland, Australia (*n* = 57; 5–15 apprentices in each group). Two of the groups were comprised of electrical apprentices and four groups were comprised of carpentry and cabinet maker apprentices. Most participants identified as male (96.49%). The focus groups were conducted by an experienced facilitator (who was also a registered psychologist), with assistance from a MATES representative. The groups were audio-recorded and transcribed verbatim. The study was approved by Griffith University's Human Research Ethics Committee (GU Reference number 2017/353).

#### Measures

The topic guide for the groups was semi-structured and used questions developed in collaboration with MATES as a general guide. Participants were asked about issues both at work and outside of work that impacted their work and their lives outside of work; the things that “stopped them from getting out of bed in the mornings”; how they manage stress when life is tough; the types of things that would be helpful when going through a tough time; and how they would help their own workmates if they were having a difficult time.

#### Data Analysis

Data was analyzed using a generic qualitative framework (18) and inductive thematic analysis (19). The approach was data-driven and the researchers identified key themes that emerged from the discussions, rather than relying on a pre-existing coding schedule. Focus group transcripts were read and coded by the first author using NVivo software. Codes and themes were discussed for consensus with the last author. Supporting quotes from the transcripts were collated for each theme and associated sub-theme.

### Quantitative Study: Online Survey

Based upon the findings of the exploratory qualitative study, the survey aimed to estimate the prevalence of workplace bullying in Queensland construction industry apprentices. Given the rates of substance use and other mental health and well-being issues within these workers, it was also important to examine bullying's associations with mental health and well-being, suicide literacy, exposure to and experience of suicidal thoughts and behaviors, perceptions of supervision quality, and stress management (including drug and alcohol use).

#### Participants and Procedure

A brief online survey was launched in August 2019 and distributed to all apprentices (~27, 000) registered in the Department of Employment, Small Business and Training (Queensland, Australia) database via a text message (20). The message contained a link to the online survey (designed to be easily completed online or via smartphone). The survey was accessible for ~8 weeks, with reminder texts sent at 2-week intervals. The study was approved by the Griffith University's Human Research Ethics Committee (GU Reference number 2019/407).

#### Measures

##### Bullying

The Negative Acts Questionnaire – Revised [NAQ-R; (21)] is a valid and reliable objective measure of bullying, consisting of 22-items that do not explicitly use the word “bullying” (e.g., “in the last 6-months, have you been humiliated or ridiculed in connection with your work”). Responses options varied from “Never” to “Daily.” For simplicity and ease of readership this will be referred to throughout as the “NAQ-R bullying scale” throughout.

##### Subjective Measure of Workplace Bullying

Participants were provided with the following statement “Workplace bullying is described as verbal, physical, social, or psychological abuse by your employer (or manager), another person or group of people at work.” Participants were then required to record how frequently they had been bullied in the previous 6-months from “Never” to “Daily.” Given low variability, responses were recoded into yes/no. For simplicity this will be referred to throughout as the “subjective bullying item.”

##### Perceptions of Supervisor

Three items were designed to capture participants' opinions of their supervisor in terms of fair treatment, respect, and clear expectations from “Strongly disagree” to “Strongly agree.”

##### Exposure to Suicide and Suicidal Thoughts

Participants were asked (yes/no) whether they whether they know someone who has attempted suicide, or whether they have known someone who has died by suicide, and whether they have personally experienced suicidal thoughts in the previous 12 months.

##### Psychological Distress

The Kessler Psychological Distress Scale [K6; (22)] was used as a brief six-item scale to assess negative emotions experienced over the preceding month (e.g., hopeless, worthless). The five-point response scale ranged from “None of the time” to “All of the time.” The K6 has demonstrated good psychometric properties and has been shown to screen for anxiety and depression (23).

##### Well-Being

The WHO-5 Well-Being Index (24) was used as a brief 5-item scale to assess positive well-being over the previous 2 weeks. The five-point response scale ranges from “At no time” to “All of the time.” The WHO-5 has been demonstrated to have excellent reliability (25).

##### Stress Management

A list of stress management activities was generated by the research team, and participants were asked the extent to which they agreed such activities helped them to manage their stress. Principal components analysis (orthogonal rotation) of items identified two factors: adaptive stress management (e.g., being with family or friends, being in nature) and maladaptive stress management (e.g., smoking, recreational drugs).

##### Drug and Alcohol Use

The AUDIT-C is a valid and highly practical brief three item screening tool (26) and was used to assess hazardous drinking behavior. Participants were also asked how regularly (if at all) they had used specified drug-related substances in the last6 months from “Never” to “Regularly.”

A final optional open-ended question was embedded in the survey for participants to voluntarily provide any additional comments.

#### Data Analysis

Following the calculation of demographic and variable frequencies, univariate logistic regressions were run to calculate odds ratios (OR) with 95% confidence intervals (95%CI) for the subjective bullying item to determine its associations with each of the demographic and psychosocial variables of interest. A series of one-way ANOVAs were conducted to compare the NAQ-R bullying scale across different demographic groups of apprentices (age, gender, industry sector, etc.) and bivariate correlations with psychosocial variables (e.g., K6, drug and alcohol use) were reported.

Sensitivity analyses using multiple imputation for missing data were conducted considering the predictor and outcome variables of interest all contained a proportion of missing data with the highest proportion of 23.5%. Fully conditional specification was used as the imputation method and Predictive Mean Matching was applied for model type with 100 iterations for maximum convergence and resulting in five imputed datasets (27, 28). Variables with binary or categorical values (such as demographic variables) were transformed into dummy-coded variables and were imputed. Categorical variables were then recreated prior to analyses on the imputed dataset.

Predictors from the previous univariate analyses (which had the significance level of 0.1) were entered into separate multivariate regression analyses with the two bullying measures as outcome variables. These variables were analyzed using the enter method rather than applying the stepwise method, which would result in different sets of variables in each imputation and make pooling estimates complicated (27). Therefore, the enter method was considered the best option to examine individual predictors associated with each bullying measure. To ensure the pooled parameter estimates in the sensitivity analyses were comparable with that of the observed (i.e., non-imputed) data, the regression model with the NAQ-R bullying scale as the outcome was performed in a linear mixed effects model and, although the subjective bullying item was a binary outcome variable, a multinomial logistic regression was run to obtain an asymptotic covariance matrix of parameter estimates needed for combining the results (28). The pooled estimates of the overall model significance (*F*-statistic) were computed using an SPSS macro by van Ginkel (29), while the pooled estimates of shared variances (*R*^2^) were computed by averaging across the *R*^2^ estimates of the regression model from imputed datasets (28). All analyses and procedures were performed in IBM SPSS Statistics software version 25.

Finally, for those participants who chose to respond to the open-ended question, written free-text responses were read and coded for themes using a similar process to the focus group transcripts described earlier. The main coder for the written responses was completely independent from the coding of the focus groups to minimize bias in results. Thematic findings were discussed with the research team for consensus.

## Results

### Qualitative Study

Analysis of the focus group data resulted in seven overarching and inter-related themes. These were (1) Bullying; (2) Variation in quality of employers/supervision; (3) Differences between older and younger workers; (4) Stress management; (5) Mental health and well-being; (6) Suicide prevention awareness training; and (7) Budgeting on a low salary. These themes and associated sub-themes are discussed in the following sections.

#### Bullying

The experience of workplace bullying perpetrated by employers and supervisors was a notable finding. This was reported by apprentices as widespread both in terms of their own personal experiences or how they witnessed the bullying of others. First year apprentices described themselves as being “at the bottom of the food chain” and accepted poor treatment as part of the workplace culture. When asked about the differences between banter and bullying, apprentices described banter as a generally harmless, while bullying was different and harmful (both were considered part of the construction industry culture). Several apprentices across the focus groups described not wanting to go to work due to their perceived poor treatment.

*There's lots of narky comments, it's not too bad… (but) when someone's really harassing you, if he's going off his nut calling you all these names and stuff, just throwing tools at you and stuff like that, then it gets a bit extreme. But that's what you have to deal with I guess*.

*At my first post… he actually put a nail gun to my head and threatened to press it - like shoot me in the head with it. That was after 6 weeks of working with this bloke, just me and him. Yeah, it was brutal*.

*There were days I didn't want to go to work just because I was stressing about the people I was going to be working with*.

##### Reluctance to Report Bullying

Apprentices generally reported that they were unwilling or unable to report bullying due to fears of retribution and adverse consequences.

*So being an apprentice we don't have any sort of right to even sort of stand up for yourself. You don't want to lose your job*.

#### Variation in Quality of Employers/Supervision

Relatedly, there was considerable variation in the quality of supervision and training described by apprentices. While some apprentices could not fault their employer, more often than not they were characterized as impatient and short-tempered with low tolerance for answering questions and any perceived lack of knowledge by apprentices (despite apprentices being in training and there to learn). Apprentices reported anxiety over not being confident in doing a job and fearing making a mistake which could prove costly. A number of apprentices mentioned how they dreaded being humiliated by their boss in front of co-workers and this had implications for their well-being and daily stress.

*When one of the tradesmen yells at you for doing something wrong, he does it in front of everyone… that also puts you down. You feel worthless*.

*I know we're apprentices and obviously we're a bit low in the food chain. But some of your good tradies will show you and actually talk to you like a human being. Then you have other ones … (where you're) just getting screamed at…*

##### Anxiety Over Moving Worksites

Consequently, many apprentices reported anxiety in changing work sites and not knowing what to expect in terms of how they will be treated and whether they will be bullied.

If you're somewhere for a week, at first you're not going to know who to go to, to ask questions. You don't know who is going to be nice to you or who is going to call you an idiot, tell you to ‘f^****^ *off’… that sort of thing*.

#### Differences Between Older and Younger Workers

Apprentices spontaneously described a difference between older employers (who were more difficult) and younger bosses (who were more patient). This was attributed to the recency of completing their own apprenticeship and a generational gap in communicating stress and emotions. Older workers were described as “old school tradies” who do not talk about their personal problems, and instead cope with their stress by taking out their anger on the apprentices. Others suggested that bullying was part of the construction industry culture and seen by older workers as a rite of passage for apprentices to endure. Apprentices also spoke of differences in communication and learning styles between the older and younger generations, with most of the opinion that older workers had very entrenched views that were unlikely to change.

*You get the older tradies who half of them feel like they need to treat you harsher because they were treated like shit when they were apprentices*.

*Well like with the tradesman I work with, because he's old, you just go “I can't make an old boy change his mind. He's going to die with his beliefs.” They're going to do what they want to do and say what they want to say, even if it's politically incorrect or whatever*.

#### Stress Management

When asked how they personally manage stress, there were a range of responses (e.g., hobbies and exercise to maladaptive stress-responses such as drinking). There was also talk of widespread drug and alcohol use across the construction industry that was seen as culturally accepted, and ranged from going to the pub to using illicit substances. Apprentices described difficulties with taking stress home to their partners and families, contributing to relationship problems and breakdowns.

*I get a bit of anxiety every now and then. If you have a crappy day, like when you're getting yelled at and stuff, you get home and you're like really ready to snap at somebody*.

*I was working with this tradesman and he's real old and he's racist, sexist, homophobic, pretty much everything under the sun. It's just like it gets to you… For me, I felt real sort of stressed out. I'd come home and be sort of aggravated. At the time I had a missus and I'd just snap over little, little things…*

#### Mental Health and Well-Being

Most apprentices reported that they would not share with their employer if they were feeling stressed, anxious, or struggling, and that they would definitely not ask for time off to deal with these problems. Several apprentices described how their employers would become angry if they took sick days and expected them to work regardless of a physical illness (e.g., influenza), and certainly not for mental health problems. Others mentioned they would take a sick leave day to deal with mental health or other issues, although they would not communicate this reason to their employer. This was largely attributed to a fear of being judged or embarrassed and was a key barrier to engaging with mental health support on site (e.g., MATES field workers). When asked about their co-workers, most apprentices expressed they could tell if someone was having a difficult time. However, despite expressing compassion for their coworkers, many communicated a reluctance to raise their concerns unless the coworker was well-known to them. In those instances, apprentices were able to describe how they had helped their close friends through hard times.

*If it is a personal reason, you have to lie and say that you're sick, and get a medical certificate, because there's no mental health days in construction, in training. I reckon half the time I call in sick, I'm not actually sick. It's like just having an off-day or whatever. Still got to get a medical certificate and say “I've got a sore throat,” or whatever (Otherwise) you'd get the sack*.

*You might not want to talk to someone about it or something but just knowing that you could (would be good)*.

*My mate, he's just broke up with his missus and he's real down. He's smoking himself silly. I just said “take it easy on yourself mate, like it's not your fault. Just because this happened and that happened it doesn't mean it doesn't happen to other people. You're not alone. Have a yarn if you need to have a yarn.”*

#### Suicide Prevention Training

There was widespread discomfort within the groups when discussing suicidal behavior (e.g., inappropriate humor, awkwardness). While admitting a lack of knowledge of suicide and how to cope with suicidal behavior (in friends and coworkers), there was also a reluctance to take on further training outside of work hours. Most apprentices agreed that suicide prevention training was important but needed it to be made a mandatory part of their apprenticeship to encourage completion.

*It's actually hard to see if the person is joking or not about suicide. You know some people are just like ‘I'm going to go kill myself’. You're like… I don't know what I'd be able to say to that. I wouldn't know what to do*.

*I've had my friends come to me and tell me “oh, this is how I am, blah, blah, blah.” I just stutter. I don't know what to say. You don't know what you can say to someone. You don't know what's going on in their head*.

#### Budgeting on a Low Salary

There was widespread mention of the difficulty of managing finances on the low salary of an apprentice. Managing the different expenses required for their training program as well as everyday expenses was difficult. Financial issues were also described within the context of negative workplace experiences, such as employers withholding adequate pay rises, being forced to work through meal breaks, and difficulties taking leave.

*When I first started, I got paid fortnightly and it was harder to budget. I found I had money one week and then I didn't the next… at the start of your apprenticeship I find people stress more about money than other things*.

### Quantitative Study

A total of 1,787 Queensland apprentices responded to the survey, indicating a response rate of 6.62%. Responses with large portions of missing data were excluded, with a final sample of *N* = 1,483 kept for analyses. Demographics for the final sample are presented in Table 1.

**Table 1:** Demographic variables and apprenticeship background (*N* = 1,483).

		***n***	**%**
Gender	Male	1,331	89.8
(*Missing: n* = 19, 1.3%)	Female	132	8.9
	Other	1	0.1
Age group	Up to 17 years	123	8.3
(*Missing: n* = 18, 1.2%)	18–25 years	873	58.9
	26–39 years	380	25.6
	40 years and over	89	6.0
Aboriginal or Torres Strait Islander	Yes	84	5.7
(*Missing: n* = 12, 0.8%)	No	1,387	93.5
Non-English speaking background	Yes	53	3.6
(*Missing: n* = 7, 0.5%)	No	1,423	96.0
LGBTI+	Yes	49	3.3
(*Missing: n* = 20, 1.3%)	No	1,414	95.3
Apprenticeship status	Active	1,203	81.1
(*Missing: n* = 5, 0.3%)	Not currently in apprenticeship	60	4.0
	Completed	183	12.3
	Ongoing but on long-term leave	16	1.1
	Other	16	1.1
Year of apprenticeship	First year	335	22.6
(*Missing: n* = 14, 0.9%)	Second year	368	24.8
	Third year	307	20.7
	Fourth year	237	16.0
	Completed within the last 12 months	181	12.2
	Other	41	2.8
Employer type	Private company	1,128	76.1
(*Missing: n* = 10, 0.7%)	Group training organization	133	9.0
	Government/Public utility	79	5.3
	Sole trader	84	5.7
	No employer currently	49	3.3
Employer size	Very large (501 or more employees)	223	15.0
(*Missing: n* = 5, 4.0%)	Large (101–500 employees)	163	11.0
	Medium (51–100 employees)	110	7.4
	Small (11–50 employees)	327	22.0
	Very small (1–10 employees)	601	40.5
Apprenticeship trade/occupation	Metal trades	162	10.9
(*Missing: n* = 17, 1.1%)	Electrical trades	362	24.4
	Plumbing trades	190	12.8
	Structural trades	413	27.8
	Finishing trades	192	12.9
	Civil and outdoor trades	37	2.5
	Construction trades	78	5.3
	Other trades	32	2.2
Highest level of education	Other apprenticeship	89	6.0
(*Missing: n* = 92, 6.2%)	University	66	4.5
	Other (e.g., TAFE, Certificate)	88	5.9
	Year 8, 9, or 10	186	12.6
	Year 11	126	8.5
	Year 12	836	56.4
Main industry sector	Workshop	182	12.3
(*Missing: n* = 13, 0.9%)	Maintenance	170	11.5
	Housing	216	14.6
	Small building/construction	262	17.7
	Commercial/Residential medium-large sites	417	28.1
	Engineering construction	38	2.6
	Civil construction	103	6.9
	Other industry	82	5.5
Overnight work in the past 12 months	None	801	54.0
(*Missing: n* = 50, 3.4%)	1–10 nights	290	19.6
	11–50 nights	198	13.4
	More than 50 nights	144	9.7

The experience of bullying was common, with nearly a third (30.8%; out of *n* = 1,322 complete responses) reporting the presence of bullying in the last 6 months (subjective bullying item), and 21.4 and 20.0% scoring within the range of occasional and severe workplace bullying on the NAQ-R bullying scale, respectively (out of *n* = 1,131). Additionally, ~13% reported elevated psychological distress (i.e., total score 19 or greater on the K6) and around 30% with reduced well-being (i.e., total score below 13 on the WHO-5). Approximately 10–12% disagreed or strongly disagreed with each of the positively worded items assessing perceptions of supervisor quality, indicating a sizeable minority felt their supervisors did not treat them fairly, nor with respect, and workplace expectations were not clear.

#### Workplace Bullying Analyses

When using the subjective bullying item, there was a significant association for apprentices who self-identified as LGBTQI+, those who were in their 2nd, 3rd, and 4th years, those who were not currently employed or worked for a group training organization, those in very large organizations, were plumbing apprentices, in the maintenance industry, and whose apprenticeships were not currently active, whereby bullying was more likely to be reported by these individuals (Table 2). Apprentices in the youngest age group (up to 17 years of age) were significantly less likely to report bullying than their older counterparts.

**Table 2:** Univariate Odds Ratios of subjective bullying in past 6-months by demographic/apprenticeship items.

		**Being bullied in the past 6 months**						
		**Yes**		**No**		**OR**	**95% CI**	
		***n***	**%**	***n***	**%**		**L**	**U**
Gender^*^	Male	361	89.6	824	90.9	0.85	0.57	1.25
	Female	42	10.4	81	8.9	1		
Age group	Up to 17 years	19	4.7	89	9.8	**0.43**	**0.25**	**0.72**
	18–25 years	254	63.0	508	56.0	1		
	26–39 years	105	26.1	250	27.6	0.84	0.64	1.10
	40 years and over	25	6.2	60	6.6	0.83	0.51	1.36
Aboriginal or Torres Strait Islander	Yes	27	6.7	49	5.4	1.27	0.78	2.06
	No	375	93.3	862	94.6	1		
Non-English background	Yes	15	3.7	31	3.4	1.10	0.59	2.07
	No	388	96.3	884	96.6	1		
LGBTI+	Yes	21	5.3	25	2.8	**1.95**	**1.08**	**3.53**
	No	379	94.8	881	97.2	1		
Apprenticeship status	Active	310	76.7	758	82.8	1		
	Not currently in apprenticeship	31	7.7	21	2.3	**3.61**	**2.04**	**6.38**
	Completed	47	11.6	122	13.3	0.94	0.66	1.35
	Ongoing but on long-term leave	8	2.0	8	0.9	2.45	0.91	6.57
	Other	8	2.0	6	0.7	**3.26**	**1.12**	**9.47**
Year of apprenticeship	First year	67	16.7	230	25.2	1		
	Second year	109	27.1	211	23.2	**1.77**	**1.24**	**2.53**
	Third year	97	24.1	180	19.8	**1.85**	**1.28**	**2.67**
	Fourth year	68	16.9	146	16.0	**1.60**	**1.08**	**2.38**
	Completed in the last 12 months	48	11.9	120	13.2	1.37	0.89	2.11
	Other	13	3.2	24	2.6	1.86	0.9	3.85
Employer type	Private company	289	71.7	729	79.8	1		
	Group training organization	43	10.7	66	7.2	**1.64**	**1.09**	**2.47**
	Government/Public utility	26	6.5	46	5.0	1.43	0.86	2.35
	Sole trader	23	5.7	49	5.4	1.18	0.71	1.98
	No current employer	22	5.5	23	2.5	**2.41**	**1.32**	**4.40**
Employer size	Very large (501+ employees)	69	17.9	126	14.2	**1.47**	**1.04**	**2.08**
	Large (101–500 employees)	50	13.0	97	10.9	1.38	0.94	2.04
	Medium (51–100 employees)	33	8.5	69	7.8	1.28	0.81	2.03
	Small (11–50 employees)	89	23.1	209	23.5	1.14	0.84	1.56
	Very small (1–10 employees)	145	37.6	389	43.7	1		
Trade	Metal trades	57	14.2	89	9.8	1.67	1.12	2.5
	Electrical trades	83	20.8	246	27.1	0.88	0.63	1.23
	Plumbing trades	66	16.5	101	11.1	**1.70**	**1.16**	**2.50**
	Structural trades	102	25.5	266	29.3	1		
	Finishing trades	56	14.0	116	12.8	1.26	0.85	1.86
	Civil and outdoor trades	11	2.8	21	2.3	1.37	0.64	2.93
	Construction trades	19	4.8	48	5.3	1.03	0.58	1.84
	Other trades	6	1.5	22	2.4	0.71	0.28	1.80
Highest education	Other apprenticeship	22	5.8	59	6.8	0.81	0.48	1.35
	University	20	5.3	42	4.8	1.03	0.59	1.79
	Other (e.g., TAFE, Certificate)	27	7.1	50	5.8	1.17	0.71	1.91
	Year 8, 9, or 10	45	11.8	126	14.5	0.77	0.53	1.12
	Year 11	29	7.6	78	9.0	0.80	0.51	1.26
	Year 12	237	62.4	512	59.1	1		
Industry sector	Workshop	60	14.9	104	11.4	1.36	0.92	2.01
	Maintenance	59	14.7	93	10.2	**1.50**	**1.01**	**2.22**
	Housing	55	13.7	138	15.2	0.94	0.64	1.38
	Small building	60	14.9	174	19.1	0.81	0.56	1.18
	Commercial/Residential	109	27.1	257	28.2	1		
	Engineering construction	12	3.0	23	2.5	1.23	0.59	2.56
	Civil construction	28	7.0	64	7.0	1.03	0.63	1.70
	Other industry	19	4.7	57	6.3	0.79	0.45	1.38
Overnight work (past 12 months)	0 nights	196	50.6	513	57.3	1		
	1–10 nights	86	22.2	181	20.2	1.24	0.92	1.69
	11–50 nights	62	16.0	116	13.0	1.40	0.99	1.98
	More than 50 nights	43	11.1	85	9.5	1.32	0.89	1.98

*N missing Subjective bullying = 161; Results in bold indicate p < 0.05; “1” = reference category;*

* *gender “other” is excluded (n = 1)*.

Table 3 presents ORs for the subjective bullying item by psychological scales and exposure to suicide. Findings indicated that the presence of bullying was significantly associated with increased drug and alcohol use, greater psychological distress, the use of more maladaptive stress management strategies, reported exposure to suicide and attempts in others, and experience of suicidal ideation within the previous 12 months. Well-being and adaptive stress management strategies were significantly negatively associated with bullying in the previous 6-months.

**Table 3:** Subjective bullying in past 6 months by psychological scales and exposure to suicide (univariate ORs).

		**Being bullied in the past 6 months**							
		**Yes**		**No**		**OR**	**95% CI**		
	***N***	***Mean* (or *n*)**	***SD* (or %)**	***Mean* (or *n*)**	***SD* (or %)**		**L**	**U**	***p*-value**
Well-being (WHO-5)	1,127	11.03	5.64	15.44	5.39	**0.87**	**0.85**	**0.89**	**<0.001**
Substance use (SU)	1,130	2.97	3.37	1.85	2.65	**1.14**	**1.08**	**1.16**	**<0.001**
Alcohol use (AUDIT-C)	1,176	5.84	3.12	5.16	3.01	**1.08**	**1.04**	**1.12**	**<0.001**
Psychological distress (K6)	1,184	16.67	5.87	11.28	4.67	**1.20**	**1.17**	**1.23**	**<0.001**
Adaptive stress management	1,181	24.41	3.42	25.13	3.41	**0.94**	**0.91**	**0.98**	**0.001**
Maladaptive stress management	984	11.48	3.96	10.28	3.66	**1.09**	**1.05**	**1.13**	**<0.001**
Exposure to suicide	1,250								
Yes		263	69.4	552	63.4	**1.31**	**1.01**	**1.70**	**0.040**
No		116	30.6	319	36.6	1			
Exposure to suicide attempt	1,265								
Yes		314	81.3	634	72.1	**1.69**	**1.25**	**2.27**	**0.001**
No		72	18.7	245	27.9	1			
Suicidal thoughts (past 12 months)	1,263								
Yes		208	54.0	235	26.8	**3.22**	**2.50**	**4.13**	**<0.001**
No		177	46.0	643	73.2	1			

*N missing in Subjective Bullying = 161*.

*Results in bold indicate significant (p <0.05); “1” = reference category*.

Results from the univariate ANOVAs and bivariate correlations with the NAQ-R bullying scale are presented in Tables 4, 5, respectively. There were significant group differences in for age group, apprenticeship status, year of apprenticeship, employer type, employer size, industry sector, and overnight work (Table 4). There were significant positive correlations between NAQ-R bullying scores and subjective bullying, substance use, problematic alcohol consumption, psychological distress, maladaptive stress management, as well as exposure to suicide and suicide attempts, and suicidal ideation. There were significant negative correlations between NAQ-R bullying scores and well-being and adaptive stress management (Table 5; Cronbach's alpha for each scale is also provided).

**Table 4:** Mean scores of self-reported bullying (NAQ-R) by demographic and apprenticeship variables.

		***n***	**Mean**	**SD**	**F**	***p*-value**
Gender^*^	Male	1,202	37.34	16.95	0.06	0.938
	Female	123	36.80	16.31		
Age group	Up to 17 years	109	32.76	16.18	**5.17**	**0.001**
	18–25 years	772	38.55	17.37		
	26–39 years	361	35.77	15.52		
	40 years and over	87	36.33	16.95		
Aboriginal or Torres Strait Islander	Yes	76	38.62	16.90	0.61	0.435
	No	1,256	37.07	16.74		
Non-English speaking background	Yes	47	36.11	14.84	0.19	0.66
	No	1,290	37.20	16.85		
LGBTI+	Yes	46	40.63	19.35	2.02	0.156
	No	1,279	37.06	16.64		
Apprenticeship status	Active	1,084	36.29	15.82	**18.10**	**<0.001**
	Not currently in apprenticeship	52	53.94	26.07		
	Completed	172	36.00	15.49		
	Ongoing but on long-term leave	16	44.69	21.09		
	Other	14	51.71	22.92		
Year of apprenticeship	First year	300	33.59	14.77	**5.69**	**<0.001**
	Second year	324	37.08	15.46		
	Third year	280	39.05	17.82		
	Fourth year	218	40.19	19.16		
	Completed within the last 12 months	170	35.81	15.70		
	Other	39	41.69	21.63		
Employer type	Private company	1,031	36.20	16.00	**9.10**	**<0.001**
	Group training organization	110	40.41	16.82		
	Government/Public utility	72	37.88	15.86		
	Sole trader	77	37.35	18.57		
	No current employer	46	50.26	25.11		
Employer size	Very large (501 or more employees)	198	38.08	15.39	**2.96**	**0.019**
	Large (101–500 employees)	150	39.65	18.57		
	Medium (51–100 employees)	102	37.82	19.36		
	Small (11–50 employees)	301	37.24	15.39		
	Very small (1–10 employees)	544	35.17	15.93		
Apprenticeship trade/occupation	Metal trades	147	39.76	17.16	1.15	0.331
	Electrical trades	334	35.71	15.65		
	Plumbing trades	171	37.46	16.69		
	Structural trades	373	37.29	17.34		
	Finishing trades	174	37.56	17.14		
	Civil and outdoor trades	33	38.52	19.41		
	Construction trades	69	34.88	15.78		
	Other trades	28	35.36	14.50		
Highest level of education	Other apprenticeship	82	35.00	13.82	0.70	0.624
	University	63	38.25	17.06		
	Other (e.g., TAFE, Certificate)	78	38.45	18.38		
	Year 8, 9, or 10	171	37.23	18.26		
	Year 11	111	35.43	16.12		
	Year 12	759	37.41	16.35		
Main industry sector	Workshop	165	40.01	17.59	**2.29**	**0.025**
	Maintenance	153	37.90	16.92		
	Housing	196	38.44	17.76		
	Small building/construction	238	35.04	15.21		
	Commercial/Residential medium-large sites	373	37.22	17.22		
	Engineering construction	36	40.08	17.19		
	Civil construction	94	35.21	15.88		
	Other industry	77	33.62	12.62		
Overnight work in the past 12 months	0 nights	721	35.74	15.64	**5.74**	**0.001**
	1–10 nights	268	36.47	14.91		
	11–50 nights	183	39.75	18.48		
	More than 50 nights	130	40.99	20.59		

*N missing in NAQ-R scale = 221; Results in bold indicate significant (p < 0.05);*

* *gender “other” is excluded (n = 1)*.

**Table 5:** Correlations between self-reported bullying (NAQ-R, Cronbach's α = 0.95) and other variables.

	***N***	**Pearson's *r***	***p*-value**	**Cronbach's α**
Subjective bullying (Yes)	1,249	0.66	**<0.001**	n/a
Well-being (WHO 5)	1,072	−0.45	**<0.001**	0.92
Substance use (SU)	1,082	0.23	**<0.001**	0.70
Alcohol use (AUDIT-C)	1,120	0.12	**<0.001**	0.70
Psychological distress (K6)	1,129	0.58	**<0.001**	0.91
Adaptive stress management	1,128	−0.11	**<0.001**	0.70
Maladaptive stress management	938	0.18	**<0.001**	0.74
Exposure to suicide (Yes)	1,188	0.09	**0.002**	n/a
Exposure to suicide attempt (Yes)	1,203	0.14	**<0.001**	n/a
Suicidal thoughts in the past 12 months (Yes)	1,200	0.36	**<0.001**	n/a

*N missing in NAQ-R scale = 221. The bold values indicate that the correlations are statistically significant (i.e. less than 0.10)*.

Table 6 displays the final models of the linear mixed-effects regression model. The overall model was significant (*F* = 12.59, *p* < 0.001). Increased bullying, measured by the NAQ-R scale, was significantly associated with greater psychological distress and with less overall well-being. Increased bullying (NAQ-R bullying scale) was also more likely among those not currently in an apprenticeship or in their 3rd year, compared to those in active apprenticeship or their 1st year of apprenticeship. Apprentices who mainly worked in an industry sector other than those listed experienced bullying less than those who worked in commercial/residential medium-larger sites.

**Table 6:** Final mixed-effects linear regression models of self-reported bullying (NAQ-R).

		**Observed data (*****N*** **= 735)**			**Sensitivity analysis (*****N*** **= 1483)**		
		**Estimate**	***SE***	***p-value***	**Estimate**	***SE***	***p-value***
Psychological distress (K6)		**1.26**	**0.13**	**<0.001**	**1.28**	**0.12**	**<0.001**
Substance use (SU)		0.35	0.20	0.077	**0.49**	**0.16**	**0.002**
Alcohol use (AUDIT-C)		−0.02	0.20	0.930	0.02	0.16	0.883
Well-being (WHO 5)		**−0.27**	**0.12**	**0.020**	**−0.30**	**0.09**	**0.002**
Adaptive stress management		0.02	0.15	0.890	−0.13	0.13	0.301
Maladaptive stress management		−0.01	0.17	0.975	−0.01	0.17	0.946
Exposure to suicide death	Yes	1.51	1.17	0.195	0.91	0.90	0.315
	No	1			1		
Exposure to suicide attempt	Yes	0.76	1.36	0.576	0.59	1.03	0.571
	No	1			1		
Suicidal thoughts	Yes	1.57	1.23	0.200	1.09	0.98	0.270
	No	1			1		
Age	Up to 17 years	−2.51	1.98	0.204	0.64	1.45	0.658
	18–25 years	1			1		
	26–39 years	−2.02	1.13	0.074	−1.59	0.87	0.068
	40 years and over	−2.19	2.22	0.324	−0.19	1.58	0.907
Employer type	Private company	1			1		
	Group training organization	−0.40	2.03	0.842	0.27	1.59	0.867
	Government/Public utility	−0.07	2.49	0.979	−0.44	1.76	0.802
	Sole trader	0.92	2.21	0.677	2.21	1.53	0.148
	No current accent	10.79	5.81	0.064	0.15	3.12	0.962
Apprenticeship status	Active	1			1		
	Not currently in apprenticeship	**16.58**	**3.34**	**<0.001**	**14.28**	**2.72**	**<0.001**
	Completed	3.29	4.73	0.486	5.44	3.13	0.083
	Ongoing but on long-term leave	7.00	5.31	0.188	5.54	1.74	0.119
	Other	3.01	5.54	0.588	5.77	1.57	0.210
Main Industry sector	Workshop	0.39	1.59	0.807	1.43	1.22	0.241
	Maintenance	−1.03	1.78	0.563	−0.69	1.40	0.626
	Housing	2.70	1.53	0.078	1.70	1.28	0.187
	Small building/construction	1.02	1.49	0.493	−0.13	1.11	0.905
	Commercial/Residential	1			1		
	Engineering construction	1.30	2.82	0.646	2.17	2.22	0.329
	Civil construction	−1.08	2.13	0.612	−0.02	1.69	0.991
	Other	**−5.06**	**2.28**	**0.027**	−2.58	1.66	0.120
Apprenticeship year	First year	1			1		
	Second year	1.55	1.43	0.279	**2.32**	**1.11**	**0.037**
	Third year	**3.54**	**1.52**	**0.020**	**4.75**	**1.17**	**<0.001**
	Fourth year	3.04	1.59	0.057	**4.87**	**1.22**	**<0.001**
	Completed (last 12 months)	−0.09	4.82	0.985	−1.29	3.18	0.685
	Other	6.59	4.00	0.100	3.37	2.62	0.203
Employer size	Very large (501+ employees)	2.75	1.78	0.122	**2.81**	**1.33**	**0.034**
	Large (101–500 employees)	0.88	1.82	0.631	**3.15**	**1.40**	**0.025**
	Medium (51–100 employees)	1.01	1.80	0.575	0.80	1.38	0.561
	Small (11–50 employees)	−0.05	1.30	0.969	1.48	0.95	0.120
	Very small (1–10 employees)	1			1		
Work overnight	0 nights	1			1		
	1–10 nights	−1.96	1.20	0.103	−0.72	0.95	0.452
	11–50 nights	1.32	1.47	0.370	**2.95**	**1.12**	**0.010**
	More than 50 nights	−0.13	1.73	0.942	2.06	1.20	0.087
		*F* = 12.59, *p* < 0.001, *R*^2^ = 41.4%			*F* = 19.71, *p* < 0.001, pooled *R*^2^ = 40.4%		

*SE = Standard Error; Results in bold indicate significant (p < 0.05); “1” = reference category*.

The pattern of results changed slightly in the sensitivity analysis including multiple imputation. Overall, the model remained significant (*F* = 19.71, *p* < 0.001). Significant associations also remained for psychological distress, well-being, those not currently in an apprenticeship, and those in their 3rd year, whereas the association for “Other” industry no longer remained significant. Several significant associations were only present in the sensitivity analysis. This included those apprentices with increased substance use, in their 2nd and 4th year, working in large or very large workplaces, and working a moderate number of overnight shifts were more likely to report bullying on the NAQ-R scale.

These findings are largely supported in the logistic regression analysis using subjective bullying as outcome. Results of the multivariate models are presented in Table 7. Again, the overall model was significant (χ^2^ = 223.28, *p* < 0.001), and greater psychological distress, those who were not currently in an apprenticeship, and those in their 3rd year were more likely to have reported subjective bullying experiences. Additionally, apprentices who worked in the plumbing trades were more likely to perceive that they had been bullied compared to those in the structural trades. Again, the sensitivity analysis using imputed data had a slightly different pattern of results. The combined overall model was significant (*F* = 4.50, *p* < 0.001). The association for plumbing apprentices was no longer significant, and significant associations emerged whereby less well-being was associated with the presence of bullying as well as for those apprentices in their 2nd year (as compared to 1st year apprentices).

**Table 7:** Final multinomial logistic regression models of subjective bullying item (Yes/No).

		**Observed data (*N* = 761)**				**Sensitivity analysis (*N* = 1,483)**			
		**OR**	**95% CI**		***p*-value**	**OR**	**95% CI**		***p*-value**
Psychological distress (K6)		**1.19**	**1.13**	**1.25**	**<0.001**	**1.16**	**1.12**	**1.20**	**<0.001**
Substance use (SU)		1.03	0.96	1.11	0.440	1.03	0.96	1.09	0.422
Alcohol use (AUDIT-C)		0.99	0.91	1.07	0.723	1.02	0.96	1.08	0.587
Well-being (WHO 5)		0.98	0.93	1.02	0.285	**0.96**	**0.93**	**0.99**	**0.022**
Adaptive stress management		0.98	0.93	1.04	0.526	0.98	0.94	1.03	0.445
Maladaptive stress management		1.02	0.96	1.09	0.489	1.02	0.97	1.08	0.425
Exposure to suicide death	Yes	1.24	0.77	1.98	0.379	1.04	0.67	1.63	0.853
	No	1				1			
Exposure to suicide attempt	Yes	0.99	0.56	1.73	0.961	0.97	0.66	1.43	0.893
	No	1				1			
Suicidal thoughts	Yes	1.07	0.67	1.69	0.782	1.01	0.71	1.43	0.948
	No	1				1			
Age	Up to 17 years	0.70	0.30	1.67	0.425	0.73	0.37	1.42	0.348
	18–25 years	1				1			
	26–39 years	1.08	0.69	1.69	0.734	0.95	0.68	1.32	0.758
	40 years and over	0.87	0.35	2.17	0.761	1.02	0.54	1.94	0.949
LGBTI+	Yes	1				1			
	No	0.33	0.10	1.06	0.062	0.49	0.24	1.01	0.052
Apprenticeship status	Active	1				1			
	Not currently in apprenticeship	**6.93**	**1.68**	**28.63**	**0.007**	**4.16**	**1.83**	**9.44**	**0.001**
	Completed	0.42	0.06	2.86	0.377	1.36	0.33	5.67	0.664
	Ongoing but on long-term leave	3.17	0.55	18.22	0.196	2.55	0.84	7.80	0.100
	Other	1.69	0.20	14.51	0.634	2.02	0.45	8.98	0.349
Apprenticeship trade/occupation	Metal trades	2.11	0.88	5.07	0.096	1.28	0.69	2.37	0.435
	Electrical trades	0.91	0.52	1.61	0.753	0.69	0.45	1.06	0.092
	Plumbing trades	**2.07**	**1.09**	**3.93**	**0.027**	1.38	0.86	2.20	0.181
	Structural trades	1				1			
	Finishing trades	1.15	0.60	2.21	0.669	0.98	0.60	1.61	0.934
	Civil and outdoor trades	2.06	0.52	8.16	0.304	1.62	0.55	4.80	0.372
	Construction trades	0.94	0.30	2.95	0.919	0.94	0.46	1.93	0.870
	Other trades	0.91	0.21	3.96	0.896	0.80	0.28	2.34	0.683
Employer type	Private company	1				1			
	Group training organization	1.14	0.52	2.51	0.747	1.41	0.82	2.42	0.212
	Government/Public utility	1.91	0.74	4.93	0.184	1.22	0.62	2.42	0.563
	Sole trader	0.70	0.28	1.75	0.451	1.31	0.69	2.50	0.411
	No current employer	1.12	0.10	12.59	0.930	0.60	0.23	1.54	0.287
Main industry sector	Workshop	0.94	0.41	2.13	0.880	1.07	0.61	1.89	0.814
	Maintenance	1.64	0.82	3.29	0.165	1.30	0.76	2.23	0.328
	Housing	1.35	0.73	2.49	0.339	1.04	0.67	1.62	0.870
	Small building/construction	1.61	0.86	2.99	0.135	0.95	0.61	1.48	0.822
	Commercial/Residential	1				1			
	Engineering construction	1.05	0.33	3.32	0.937	1.07	0.44	2.62	0.882
	Civil construction	1.24	0.48	3.23	0.656	1.13	0.58	2.19	0.718
	Other	0.64	0.24	1.68	0.366	0.83	0.42	1.63	0.584
Apprenticeship year	First year	1				1			
	Second year	1.74	0.97	3.13	0.064	**1.60**	**1.03**	**2.49**	**0.038**
	Third year	**2.11**	**1.14**	**3.91**	**0.017**	**1.82**	**1.17**	**2.82**	**0.008**
	Fourth year	1.27	0.65	2.45	0.483	1.36	1.36	0.84	0.209
	Completed (last 12 months)	5.71	0.83	39.25	0.077	1.18	0.29	4.70	0.816
	Other	3.43	0.69	16.95	0.131	1.26	0.48	3.26	0.637
Employer size	Very large (501+ employees)	1.50	0.74	3.07	0.264	1.44	0.85	2.44	0.171
	Large (101–500 employees)	1.03	0.51	2.09	0.939	1.09	0.64	1.86	0.747
	Medium (51–100 employees)	0.92	0.44	1.90	0.818	1.16	0.68	1.98	0.588
	Small (11–50 employees)	1.08	0.65	1.79	0.775	1.11	0.76	1.64	0.585
	Very small (1–10 employees)	1				1			
Work overnight	0 nights	1				1			
	1–10 nights	1.10	0.69	1.76	0.682	1.06	0.73	1.54	0.750
	11–50 nights	1.24	0.71	2.17	0.454	1.30	0.87	1.94	0.196
	More than 50 nights	0.80	0.41	1.59	0.524	1.01	0.62	1.63	0.977
		χ^2^ = 223.28, *p* < 0.001,				*F* = 4.50, *p* < 0.001,			
		Nagelkerke's *R*^2^ = 35.6%				Pooled Nagelkerke's *R* = 29.5%			

*Results in bold indicate significant (p <0.05); “1” = reference category*.

Thematic analysis of responses (*N* = 160) to the optional open ended question “Do you have any other comments” identified five overarching themes: lack of financial security, bullying and harassment, work pressures, lack of support for training/education, and stigma toward mental health within the construction industry. These themes revealed a similar pattern of results to the qualitative study.

## Discussion

Young men working in the construction industry are more likely to die by suicide (2), and experience adverse working conditions such as routine workplace bullying (12–14). Our study is novel in that it used an exploratory sequential mixed methods design to examine the factors associated with the mental health and well-being of construction industry apprentices in Queensland, Australia. Consistent with this design, the current study aimed to first explore the well-being of apprentices in a qualitative study. Workplace bullying emerged as a key experience that had adverse implications for the mental health and well-being of apprentices. A subsequent quantitative study was developed to determine the prevalence and associated psychosocial features of workplace bullying in construction industry apprentices, including exposure to suicidal thoughts and behaviors, so as to inform future prevention program development.

The experience of workplace bullying was commonly described by construction apprentices in our focus groups and highlighted as entrenched in the worksite and industry culture. Workplace bullying was reported to be associated with a range of co-occurring issues that have ongoing implications for the well-being of apprentices, their families, and their careers. A clear observation was the reluctance of apprentices to report their poor treatment or seek help, as also seen in a previous qualitative study from Australia (12). While not unique to apprentices in construction (30), the poor treatment of new trainee workers with limited access to support coincides with high rates of substance misuse (e.g., 32), and suicide (1–6) in this industry in particular. This suggests the indoctrination and perpetuation of a workplace culture that prioritizes stoicism (11) via bullying, intimidation, and difficult working conditions may be associated with adverse mental health consequences (13). This was explored further in the quantitative component of this study.

To the best of our knowledge, the quantitative component of our mixed methods study presents the largest cross-sectional survey to examine workplace bullying and associated factors in Australian construction industry apprentices to date. Two measures were used to assess bullying. The subjective bullying item was used to determine, from the perspective of the apprentices themselves, whether they felt bullied in the workplace according to a predetermined definition. The NAQ-R was used to measure exposure diverse workplace experiences from not exposed at all to highly exposed, as opposed to a simple yes/no response (21). It has been recommended in the literature to utilize both the self-labeling subjective perception of bullying as well as using a valid and reliable scale (21). Consistent with the qualitative component of our study, the survey revealed that workplace bullying was a relatively common experience, with 30% self-labeling themselves as a victim of workplace bullying using the subjective item, and 20% scoring within the severe range on the NAQ-R bullying scale. The survey also allowed exploration of whether certain types of apprenticeships or workplace experiences were more likely to experience bullying, and whether bullying was associated with poorer psychosocial outcomes and suicidality. Greater psychological distress, being in their 3rd year, or not currently active in their apprenticeship were each consistently associated with *both* bullying measures across the multivariate models. Poorer well-being was significant in both models for the NAQ-R bullying measure only. This aligns with previous research whereby workplace bullying is associated with apprenticeship non-completion in the construction, engineering and hospitality fields (15), and made more likely given the tenuous nature of construction work (14) and the propensity for workplace victims to avoid rather than act in these situations (31). A previous small-scale survey of 169 Australian construction apprentices also found similar associations between psychological distress and workplace bullying (13).

Our sensitivity analyses for both bullying measures showed some deviation and we need to be cautious when interpreting some results. For the subjective bullying item, poorer well-being and being a 2nd year apprentice were significant only in the imputed model, whereas the association for plumbing apprentices did not retain significance. For the NAQ-R bullying measure, greater substance use, being a 2nd or 3rd year apprentice, working for large or very large organizations, and working nights away from home were significant in the sensitivity analysis, and working in a non-listed industry sector was significant only in the initial model. These findings provide an indication of possible workplace experiences (e.g., plumbing, working away from home) where bullying may be problematic. Considering the differential findings across the sensitivity analysis, conclusions drawn from these results must be taken cautiously with additional research required (32); however, they are somewhat supported given previous research has similarly reported an association with workplace bullying and substance use in the construction industry (13).

Exposure to others' suicide death and suicide attempts, and personal suicidal ideation were each associated with both measures of bullying in the univariate analyses; however, these were no longer significant in the multivariate models. Given the vulnerability of workers in this industry to be exposed to suicidal behaviors, it is crucial that future research examines whether the associations between routine workplace bullying and suicide related behaviors are mediated by other factors such as psychological distress. Other areas for future research include the development and refinement of workplace strategies to reduce the impact of bullying in these vulnerable workers. For workplace bullying, the tendency to use avoidance coping (13) necessitates a prevention approach (33). Future research is therefore required to better understand the top-down and bottom-up processes between tradespeople/supervisors and apprentices, workplace culture, and the role of workplace and financial stress in order to inform a tailored prevention strategy.

It is interesting that the results suggest that 1st year apprentices, who would be expected to be the most vulnerable due to their young age, were less likely to be bullied. It is possible that this group may be more protected (as many commence their apprenticeships while still attending school), that supervisors may be more demanding of older apprentices, or perhaps new apprentices do not recognize certain behaviors as bullying.

Several limitations to the current study should be noted. These include the reliance on electrical and carpentry apprentices in the focus groups, forgoing the inclusion of perspectives from other industry sectors that may be more vulnerable (e.g., plumbing). It was also not possible to cross-validate the qualitative findings with focus groups participants. For the survey, there was a relatively low response rate which may have introduced bias into the results. Finally, as the impact of workplace bullying is likely to unfold over time, the use of a cross-sectional design limits our ability to determine causation.

The results of this study indicate that a substantial proportion of construction industry apprentices experience workplace bullying, are exposed to suicidal behaviors, and personally experience suicidal ideation. Analyses showed that both subjective and objective measures of bullying were associated with greater psychological distress, as well as being a 3rd year apprentice, or not currently in an active apprenticeship. There were also indications that bullying may be associated with substance use, lower levels of well-being, working nights away from home, the plumbing trades, and working for larger organizations. The implications of these results should not be underestimated. Given the vulnerability of apprentices and the strong industry support to address the issue of workplace bullying, the timely development of an appropriate intervention will be critical. The study findings will be fundamental for informing the development of policies and an evidence-based intervention to address bullying and the mental health of apprentices in this sector. It will be important that these interventions are tailored to the needs of the industry and facilitate workplace cultural change among supervisors, colleagues, and apprentices alike. Such interventions may also contribute to alleviating related issues such as substance use and poor well-being.

## Data Availability Statement

The raw data supporting the conclusions of this article will be made available by the authors, without undue reservation.

## Ethics Statement

The studies involving human participants were reviewed and approved by Griffith University Human Research Ethics Committee (GU Reference number 2019/407). Written informed consent from the participants' legal guardian/next of kin was not required to participate in this study in accordance with the national legislation and the institutional requirements.

## Author Contributions

VR, JG, and KK: conceptualization. VR and KK: methodology and supervision. VR and SM: qualitative analysis and writing – manuscript. RW: quantitative analysis. VR: writing – original reports. VR and JG: project administration and funding acquisition. All authors writing – review and editing. All authors have agreed to the final version of the manuscript.

## Conflict of Interest

The authors declare that the research was conducted in the absence of any commercial or financial relationships that could be construed as a potential conflict of interest.
